# Quantitative proteomic analysis shows involvement of the p38 MAPK pathway in bovine parainfluenza virus type 3 replication

**DOI:** 10.1186/s12985-022-01834-x

**Published:** 2022-07-13

**Authors:** Liyang Li, Pengfei Li, Ao Chen, Hanbing Li, Zhe Liu, Liyun Yu, Xilin Hou

**Affiliations:** 1 Heilongjiang Bayi Agricultural University, Daqing, 163319 China; 2 Daqing Center of Inspection and Testing for Rural Affairs Agricultural Products and Processed Products, Ministry of Agriculture and Rural Affairs, Heilongjiang Bayi Agricultural University, Daqing, 163319 China; 3 Department of Nephrology, Fifth Affiliated Hospital of Harbin Medical University, Daqing, 163319 China

**Keywords:** Bovine parainfluenza virus type 3 (BPIV3), Differentially expressed proteins, p38 MAPK signaling pathway, Quantitative proteomics

## Abstract

**Background:**

Bovine parainfluenza virus type 3 (BPIV3) infection often causes respiratory tissue damage and immunosuppression and further results in bovine respiratory disease complex (BRDC), one of the major diseases in dairy cattle, caused huge economical losses every year. However, the pathogenetic and immunoregulatory mechanisms involved in the process of BPIV3 infection remain unknown. However, the pathogenetic and immunoregulatory mechanisms involved in the process of BPIV3 infection remain unknown. Proteomics is a powerful tool for high-throughput identification of proteins, which has been widely used to understand how viruses interact with host cells.

**Methods:**

In the present study, we report a proteomic analysis to investigate the whole cellular protein alterations of MDBK cells infected with BPIV3. To investigate the infection process of BPIV3 and the immune response mechanism of MDBK cells, isobaric tags for relative and absolute quantitation analysis (iTRAQ) and Q-Exactive mass spectrometry-based proteomics were performed. The differentially expressed proteins (DEPs) involved in the BPIV3 invasion process in MDBK cells were identified, annotated, and quantitated.

**Results:**

A total of 116 proteins, which included 74 upregulated proteins and 42 downregulated proteins, were identified as DEPs between the BPIV3-infected and the mock-infected groups. These DEPs included corresponding proteins related to inflammatory response, immune response, and lipid metabolism. These results might provide some insights for understanding the pathogenesis of BPIV3. Fluorescent quantitative PCR and western blotting analysis showed results consistent with those of iTRAQ identification. Interestingly, the upregulated protein MKK3 was associated with the p38 MAPK signaling pathway.

**Conclusions:**

The results of proteomics analysis indicated BPIV3 infection could activate the p38 MAPK pathway to promote virus replication.

## Introduction

Bovine parainfluenza virus type 3 (BPIV3) is an enveloped, single-stranded negative-sense RNA virus that belongs to the family Paramyxoviridae, genus Respirovirus [1]. BPIV3 infection results in pneumonia and atypical interstitial pneumonia in cattle and leads to severe secondary bacterial infection and other related clinical symptoms. BPIV3 infection and other viral or bacterial infections often cause bovine respiratory disease complex (BRDC) [2]. The cattle mortality by BRDC is up to 35%, which causes huge economic losses in the cattle industry [3]. The genome of BPIV3, approximately 15 kb in size, encodes six structural proteins and three nonstructural proteins [4, 5]. The structural proteins include nucleoprotein (N), phosphoprotein (P), large protein (L), matrix protein (M), hemagglutinin-neuraminidase (HN), and the homotrimeric fusion (F), while the accessory nonstructural proteins include C, V, and D proteins. Multiple functions and activities of the structural and accessory proteins have been investigated. For example, the glycoprotein HN binds to the receptor protein on the host cell surface, followed by the fusion protein F to induce membrane fusion [6, 7]. The conserved, nonglycosolated matrix protein (M), is the most abundant viral protein in an infected cell. The nonstructural proteins including V protein and C protein are also encoded by the P gene. The V, C, and N proteins together regulate virus replication [5]. Although much progress has been made in understanding the proteins of BPIV3, the pathogenetic and immunoregulatory mechanisms involved in the process of BPIV3 infection remain largely unclear. To investigate the changes in the host physiological system during the process of viral invasion, isobaric tags for relative and absolute quantitation analysis (iTRAQ) mass spectrometry (MS)-based global proteomics profiling was performed.

The iTRAQ quantitative proteomics technique has been widely used to study interaction between virus and host based on high sensitivity and quantitation accuracy [8]. An et al. used iTRAQ to determine the differentially expressed proteins (DEPs) of transmissible gastroenteritis virus (TGEV)-infected PK-15 cells, which identified 60 upregulated and 102 downregulated proteins in the TGEV infection process. Their analysis revealed that many upregulated proteins were associated with interferon signaling and that TGEV infection could activate the JAK-STAT1 signaling pathway [9]. In order to provide a scientific basis for the PEDV pathogenesis, the iTRAQ quantitative proteomics technique identified the proteins associated with porcine epidemic diarrhea virus (PEDV) infection [10]. Isobaric tags for relative and absolute quantification (iTRAQ) combined with liquid chromatography-tandem mass spectrometry (LC-MS/MS) approaches have been used to provide the proteomic expression profiles of host cells in response to infections by various viruses, including classical swine fever virus [11], porcine deltacoronavirus [12], influenza A (H1N1) virus [13], and porcine rotavirus [14]. iTRAQ coupled with LC-MS/MS analysis is a robust quantitative proteomics technique for the comprehensive analysis of differentially expressed proteins (DEPs). In the present study, the DEPs in BPIV3-infected MDBK cells were identified and quantitatively analyzed for the first time by the iTRAQ-based proteomics approach. MDBK cells have been selected for use in many studies [15, 16]. Usually MDBK cells are not only used for the isolation, propagation, and basic studies of BPIV3, but also as host many other bovine pathogens, such as bovine respiratory syncytial virus (BRSV) and bovine herpesvirus type 1 [17, 18].

The expression levels of 116 proteins were found to be significantly altered after 24 h of BPIV3 infection. These cellular DEPs were assigned to several biological processes according to bioinformatics analysis. These changes activated the p38 MAPK pathway promoted the BPIV3 replication, providing a global understanding of the host action with BPIV3 infection.

## Materials and methods

### Virus infection of MDBK cells

MDBK cells were cultured in DMEM (Dulbecco’s modified Eagle’s medium) medium containing 10% fetal bovine serum (FBS) and 100 g /ml penicillin and 100 g /ml streptomycin. Cell culture conditions at 37 °C with 5% CO_2_ in 24 h. The BPIV3 DQ strain (GenBank accession no. HQ462571) was isolated and identified in the preventive veterinary laboratory of Heilongjiang Bayi Agricultural University. MDBK cells were infected with BPIV3 at multiplicity of infection (MOI = 1). Uninfected cells were used as mock-infected groups. Each experiment was carried out with three replicates. The cytopathic effect (CPE) was observed and the growth curve of BPIV3 was measured. TCID_50_ were measured by the Reed-Muench method.

### Protein isolation, digestion, and labeling with iTRAQ reagents

All the cell samples, including BPIV3-infected group and control group, were cleaned with cold PBS twice and centrifuged at 1000 g at 4 °C for 10 min to harvest cells. Then, the collected cells were lysed to extract proteins in the 300 μL SDT (1 mM PMSF, 2 mM EDTA and 10 mM DTT). The dissolved protein samples were harvested with centrifugation at 1 4000 g for 40 min at 4 °C. The concentration of the protein supernatant was determined using BCA protein assay. The protein 100 μg was digested for 8 h at 37 °C by the sequencing-grade modified trypsin. The protein samples were labeled by different iTRAQ tags on the basis of iTRAQ Reagent-8plex Multiplex Kit instruction (AB SCIEX). Three mock-infected samples were labeled by iTRAQ 113, iTRAQ 114 and iTRAQ 115, respectively; three BPIV3-infected samples were labeled by iTRAQ 116, iTRAQ 117 and iTRAQ 118, respectively. Then the labeled samples were mixed and dried by using vacuum concentrator.

### LC–MS/MS analysis

The labeled peptide samples were purified and separated by AKTA purification system. The operation methods and solution preparation were performed essentially as described previously [19]. The whole elution process was monitored at 214 nm and collected every minute. Thirty distillates were collected and neutralized in 10 pools and desalinated in a C18 cartridge. After each fraction was vacuum centrifuged, the sample was dissolved in 40 μL 0.1% trifluoroacetic acid and kept frozen at − 80 °C for mass spectrometry analysis. Each sample was separated by capillary high-performance liquid chromatography (Thermo scientific EASY column (2 cm, 100 μm 5 μm, C18). The chromatography conditions were as follow: Water with 0.1% formic acid (A) and Acetonitirile with 0.1% formic acid (B) as mobile phase. The flow rate was 300 nL per minute and the mobile phase gradient program was used: 0–33 min, from 0 to 40%(B); 33–34 min, from 40 to 100%(B); 34–35 min maintained 100% and then back to 40%. Then, proteins were analyzed by using a Q-Exactive mass spectrometry (Thermo Finnigan) at positive ion mode (parameters: mass range: 300–1800 m/z; Dynamic exclusion: 40.0 s, MS2 Activation Type: HCD, Normalized collision energy: 30 eV).

### Database search and bioinformatic analysis

MS/MS data were searched in the bovine subset database from the UniProt database (release March 22, 2016, containing 32 015 sequences) and proteins were identified by Mascot 2.3.02. The peptide for quantification was automatically selected by Paragon™ algorithm to calculate the reporter peak area, error factor (EF) and p-value. The proteins expression levels in BPIV3-infected cells were calculated to compare with those of mock-infected cells. Proteins with fold changes > 1.5 and p-values < 0.05 were considered as significantly different expressions. Auto bias-corrected were executed to decrease artificial error. These proteins were further classified by Gene Ontology (GO) and pathway enrichment analysis (http://www.geneontology.org).

### RNA extraction and real-time PCR analysis

The mRNA levels of differentially expressed proteins were analyzed by real-time PCR. Total RNA of the MDBK cells in the BPIV3 infected group and the control group was extracted by TRIzol reagent (Takara) according to the manufacturer’s protocol. The RNA concentration was measured using NanoDropnd-1000. Agarose gel electrophoresis detected the total RNA 1 μL. The cDNAs of these samples were obtained by reverse transcription. Relative quantitative real-time PCR was performed in a 25 μL system that containing 12.5 μL SYBR Premix Ex TaqTM II, 2 μL primers, 2 μL cDNA samples and 8.5 μL water. The reaction condition was 95 °C for 10 min, then 40 cycles of 95 °C for 30 s, 57 °C for 30 s and 72 °C for 30 s. The melting curves were obtained. The gene of GADPH was used as the internal reference gene. All of the primers were used in the PCR tests shown in Table 1. The data statistic was based on three independent experiments.

**Table 1:** The primers of genes (5′→3′)

Gene name	Acc. number	Primer	Sequence	Length
*MHCII*	Q9TTM7	Fwd	GAGCGAGTGTCATTTCTTCAAC	22
		Rev	GCACGAACTCTTCTCCATTATG	22
*GSTA1*	A5PJE0	Fwd	TCCAAGAGAGGGCAACAAAC	20
		Rev	TCCACATAATAGAGCAATTCAACC	24
*MKK3*	A4IFH7	Fwd	TGAAGCAGGTGGTAGAGGAG	20
		Rev	CACGAAGGCAGCGATGTC	18
*AP-2*	P63009	Fwd	GTGATTGCTGCTATGACTGTG	21
		Rev	ATGTCTGGCTGACTCTTGG	19
*MARCS*	P12624	Fwd	CTACAGTGCGGCTACAAATC	20
		Rev	TGAAGAGGACAGAACAGAACC	21
*Sep*	P49907	Fwd	GCTGGCTCTGGCTCTCTG	18
		Rev	GGTGGAGGTTGCTTACAATAGG	22
*FGF13*	BC149415	Fwd	GCTGAACGGAGGCAAGTC	18
		Rev	TGATGGCAGATTAGAATAGTGAAC	24
*TFPI*	Q7YRQ8	Fwd	GGCTGTGTTCTGCTAATGTC	20
		Rev	AGTCTTGGCATCTTCTTGTTC	21
*GADPH*	AB098979	Fwd	TTCAACGGCACAGTCAAGG	19
		Rev	CTCAGCACCAGCATCACC	18

Fwd indicates the forward primer; Rev indicates the reverse primer

### Western-blot

The infected MDBK cells were washed two times with PBS and disrupted with lysis buffer (50 mM Tris–HCl, pH 8.0, 150 mM NaCl and 1% Triton X-100, supplemented with 1 tablet of Complete-Mini Protease Inhibitor Cocktail per 50 ml buffer). The cell lysates were centrifugated at 12,000 × g for 10 min to harvest supernatants. Protein assays were performed on all supernatants using the Bradford method. For Western blot analysis of the whole-cell lysates, samples, each containing 25–30 μg of protein equivalent, were dissociated in SDS-PAGE loading buffer and separated by 12% gradient SDS-PAGE. Proteins were then transferred to an Immobilon-FL membrane (Millipore). The primary antibodies, including MKK3 (rabbit, Cell Signal Technology5674, Danvers, MA), p38 phosphorylation (p-38) 1:1000 (mouse, Cell Signal Technology9216, Danvers, MA), p-38 1:1000 (rabbit, Cell Signal Technology41666, Danvers, MA), β-actin 1:10,000 (mouse, Sigma), were incubated on the membrane at 4 °C over-night. As a secondary antibody, goat anti-rabbit and goat anti-mouse immunoglobulin G (1:1000, Santa Cruz Biotechnology Inc.) was applied at room temperature for 1 h. After further washes, the immune complexes were revealed by enhanced chemluminescence by the ECL detection kit (Beijing Biosea Biotechnology Co., Ltd.).

### Statistical analysis

Statistical analysis was performed in Microsoft Excel for two-tailed Student’s t test or one-way analysis of variance (ANOVA). The p-values < 0.05 were considered statistically significant.

## Results

### Detection of theBPIV3 activity in MDBK cells

**Fig. 1:**
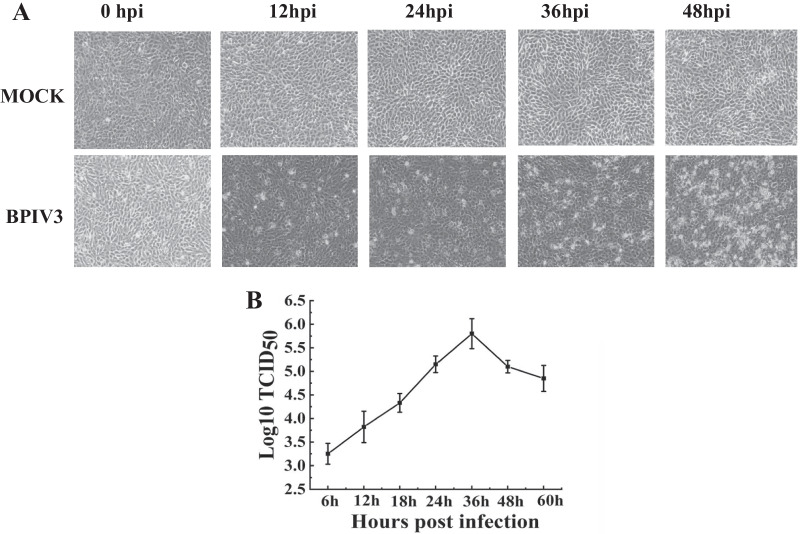
Virus infection. **A** Photomicrographs of MDBK cells infected with BPIV3 strain DQ at MOI = 1 or mock-infected at different times after infection. Images were taken at an original magnification of 40×. **B** One-step growth curve of BPIV3 strain DQ in MDBK cells

To determine the optimal sampling time point for proteomics analysis after BPIV3 infection, MDBK cells were cultured in a monolayer and inoculated with BPIV3. At different time points 0, 6, 12, 18, 24, 36, and 48 h post inoculation, the cell-virus suspension was harvested and the CPE was observed (Fig. 1A). The TCID_50_ was measured. The growth curve of BPIV3 was plotted according to the results of TCID_50_ which showed that BPIV3 proliferated rapidly from 24 to 36 h after infection, indicating active intracellular replication of the virus (Fig. 1B).

The MDBK cells were inoculated with BPIV3 at the dose of 1 multiplicity of infection (MOI = 1), and CPE was observed at different time points after infection. The results showed that lesions began apparently at 12 h after BPIV3 infected the cells, and then, became more worse with time (Fig. 1A). The viral titer reached a peak of approximately 5.7 at 36 h and then gradually and continuously declined (Fig. 1B). Generally, the optimal time for a proteomic analysis is when viral replication remains high but no significant host cell cytoskeleton or membrane rearrangement is observed [20]. According to the post-infection cytopathic conditions combined with virus proliferation, cells infected at 24 h were used as the time point for proteomics analysis.

### Protein profiling and iTRAQ quantification

The collected protein samples of BPIV3-infected and mock-infected MDBK cells were labeled with iTRAQ reagent in three biological replicates. The quantitative information of the two experimental group ratios (ratio [infection/control]) was obtained by integrating the peptide segment information of three biological duplicates in the mock-infected group (control) and the BPIV3-infected group (infection).

**Fig. 2:**
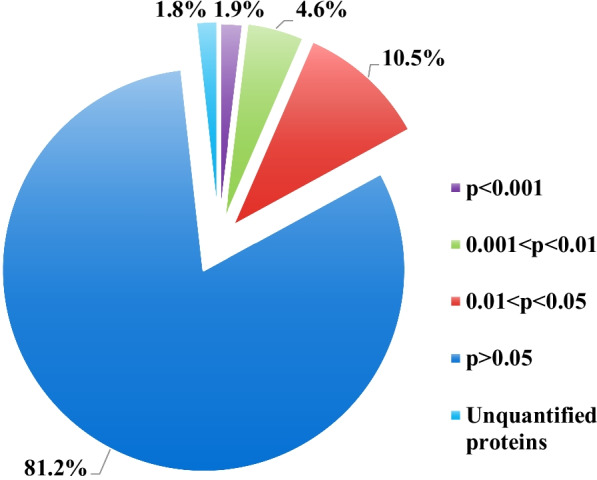
The quantitation and significance analysis of the 2804 identified proteins from BPIV3-infected and mock-infected groups

The changes in the protein expression level between the two groups were analyzed based on statistical significance. A total of 2804 proteins were detected and quantified by LC–MS/MS. 116 proteins significantly changed according to *P* < 0.05 (Fig. 2) and the proteins change ratio of ≥ 1.5. Among these proteins, 74 proteins were significantly upregulated and 42 proteins were markedly downregulated (Table. 2). The most significantly upregulated protein among the DEPs was vesicle-related membrane protein, which is related to autophagy. The most significantly downregulated protein was the integrin complement protein, which is a receptor protein of viral infection (Table. 2).

**Table 2:** The DEPs lists betweenBPIV3-infected group and mock group

No.	Protein name	Uniprot Accession no	GO annotation			P value	V/C
			Biological process	Cell component	Molecular function		
1	Integrin beta	Q6PT99	Single organismal cell–cell; adhesionresponse to stimulus	Plasma membrane region	Integrin binding; molecular transducer activity; receptor activity	0.042	0.412
2	Uncharacterized	E1BEW4	Regulation of cellular component organization	Membrane	protein kinase activity; purine nucleotide binding	0.014	0.421
3	OCRL protein	A7E337	Cellular component assembly; regulation of metabolic process	Extracellular region part; cytoplasm	Phosphatase activity; GTPase binding;	0.037	0.425
4	DTYMK protein	A5PJV9	Metabolic process; biosynthetic process;	Cytoplasm	Nucleotide kinase activity;	0.034	0.497
5	Myosin-7	Q9BE39	Biosynthetic process; metabolic process;	Actin cytoskeleton; intracellular	Binding	0.032	0.409
6	Uncharacterized protein	E1BF95	Regulation of kinase activity;regulation of metabolic process	Intracellular	Purine nucleotide binding	0.048	0.420
7	Mesoderm induction early response protein 2	A5PJX4	Regulation of primary metabolic process; regulation of cellular biosynthetic process	Nucleus; cellular_component	Binding	0.037	0.466
8	Uncharacterized protein	E1BKT3	Macromolecule localization; intracellular transport	Plasma membrane region; Membrane-	Integrin binding	0.037	0.477
9	Selenoprotein P	P49907	Signal transduction; G-protein coupled receptor signaling pathway	Plasma membrane region; Dendrite	Signaling receptor activity;	0.042	0.480
10	ERGIC and golgi 2	Q0IIJ1	–	Intracellular organelle; membrane-bounded organelle	–	0.044	0.483
11	Uncharacterized protein	G5E5P7	–	Cytoskeleton; intracellular part	–	0.012	0.516
12	N-acylglucosamine 2-epimerase	G3MZ53	Small molecule metabolic process	–	Racemase and epimerase activity	0.006	0.524
13	Serine protease HTRA1	F1N152	Regulation of metabolic process; Signal transduction	Cytoplasm; extracellular matrix	Serine hydrolase activity; insulin-like growth factor binding	0.002	0.527
14	GTP-binding protein SAR1a	Q3T0D7	Macromolecule localization; cellular component assembly	Endoplasmic reticulum; membrane-bounded vesicle	Small molecule binding;	0.049	0.528
15	Uncharacterized protein	F1MDD5	Developmental process; cell differentiation	Extracellular organelle	Receptor binding	0.007	0.529
16	Clusterin	F1MWI1	Cell death; cellular process	–	–	0.002	0.533
17	Rab5 GDP/GTP ex-change factor	O18973	Regulation of metabolic process; Regulation of transport	Cytoplasm; Recycling endosome	Regulation of metabolic process	0.022	0.541
18	Calcyphosin	Q0VCC0	Metabolic process; regulation of cellular catabolic proces	cellular_component; intracellular part	Cation binding	0.001	0.558
19	Uncharacterized protein	F1MS35	Cellular component assembly; positive regulation of metabolic process	cytoplasmic vesicle; nucleus	Cation binding; enzyme regulator activity	0.010	0.560
20	Tissue factor pathway inhibitor 2	Q7YRQ8	Regulation of metabolic process;circulatory system development	Membrane-bounded organelle;	Endopeptidase regulator activity	0.000	0.563
21	TATA box-binding protein-associated factor RNA polymerase I subunit D	Q32LB6	Regulation of metabolic process	Membrane-bounded organelle	Nucleic acid binding	0.031	0.566
22	Uncharacterized protein	E1BDC9	Digestive tract development; cell differentiation	Cytoplasm; end-omembrane system	binDing	0.032	0.570
23	Uncharacterized protein	F1N2K8	Single-organism developmental process	Cytoplasmic part	Protein binding	0.007	0.578
24	Uncharacterized protein	F1MH50	Regulation of cytoskeleton organization	Cytoplasm	Protein binding	0.001	0.579
25	Uncharacterized protein	G3N1L7		Extracellular matrix	Ion binding	0.004	0.582
26	MHC(BoLA) class II DR-beta chain	Q9TTM7	Antigen processing and presentation	Extracellular matrix;cytoplasmic part	Binding	0.033	0.582
27	Transmembrane protein 106B	Q3ZC25	Developmental process; Cell morpho-genesis	Membrane; Cytoplasm	–	0.002	0.582
28	Uncharacterized protein	E1BM92	Single-multicellular organism process	Plasma membrane region; Cytoplasm	Cytoskeletal protein binding	0.041	0.588
29	Mitochondrial ribonuclease P protein 1	Q2KI45	Nucleic acid metabolic process	Intracellular organelle; Nucleoplasm	Catalytic activity; Transferase activity	0.033	0.594
30	Periplakin	M5FKH8	Developmental process;	Cytoskeleton; Intracellular part	Binding	0.004	0.595
31	Proteasome assembly chaperone 1	Q0P5F2	Cellular component assembly; Organ development	Cytoplasm; Intracellular organelle	Proteasome binding	0.035	0.597
32	Uncharacterized protein	E1B8X6	Transport;	Intracellular; Lysosome	–	0.009	0.607
33	Glutathione S-transferase	A5PJE0	Metabolic process	intracellular part	Transferase activity	0.016	0.607
34	Receptor-type tyrosine-protein phosphatase F	A7MBJ4	Regulation of response to stimulus; cell development	Membrane	Anion binding	0.025	0.608
35	Haloacid dehalogenase-like hydrolase domain-containing protein 3	Q5E9D6	Small molecule metabolic process	–	Hydrolase activity; phosphatase activity	0.015	0.612
36	Uncharacterized protein	F1MD78	–	Intracellular organelle part; nuclear part	–	0.015	0.614
37	Alpha-1-antiproteinase	P34955	Regulation of metabolic process; Negative regulation of catalytic activity	Endoplasmic reticulum; intracellular organelle	Glycoprotein binding; enzyme binding; enzyme inhibitor activity	0.044	0.617
38	NUP35 protein	A6QPZ3	Transport	Membrane	–	0.010	0.619
39	Lysosomal alpha-glucosidase	Q9MYM4	Metabolic process	Intracellular organelle; extracellular organelle	Hydrolase activity	0.025	0.625
40	Uncharacterized protein	F1MJB0	–	–	–	0.013	0.626
41	Uncharacterized protein	E1BI31	Positive regulation of immune system process	Intracellular organelle; membrane	Enzyme binding	0.040	0.629
42	Proteasome subunit alpha type-2	Q3T0Y5	Cellular protein metabolic process; multi-organism process	Extracellular vesicle; intracellular organelle	Endopeptidase activity	0.031	0.631
43	Uncharacterized protein	F1MNT4	tissue development; regulation of cellular process	Extracellular matrix	Integrin binding	0.002	1.502
44	Up-regulator of cell proliferation	G3X839	apoptotic process	–	–	0.011	1.504
45	Uncharacterized protein	F1MPD4	–	–	–	0.000	1.508
46	Uncharacterized protein	G3MZ27	Single-organism process	Membrane part	Trans-membrane signaling receptor activity	0.003	1.511
47	CD44 antigen	F1MHC3	Cell adhesion	Integral component of membrane	Binding	0.000	1.512
48	Uncharacterized protein	G3X6B3	Immune system process	Membrane	Nucleic acid binding	0.041	1.515
49	Leucine-rich repeat flightless-interacting protein 2	E1BBW0	–	Cytoplasm; membrane	Protein binding	0.009	1.530
50	Tyrosine-protein phosphatase non-receptor type	A6QQN2	Apoptotic signaling pathway	Endoplasmic reticulum	Phosphatase activity	0.006	1.537
51	Uncharacterized protein	G5E6P8	Lipid metabolic process	Membrane	Hydrolase activity	0.016	1.537
52	Mitogen-activated protein kinase 7	A5PKJ4	Intracellular transport	Cytoplasm	Kinase binding	0.044	1.541
53	78 kDa glucose-regulated protein	Q0VCX2	Regulation of cell migration	Cytoplasmic vesicle	Small molecule binding	0.000	1.542
54	NSL1 protein	A6QQ16	Primary metabolic process	Intracellular organelle	Hydrolase activity	0.037	1.544
55	Tryptophan-tRNA ligase, cytoplasmic	P17248	Metabolic process	Intracellular organelle	Binding	0.000	1.544
56	Uncharacterized protein	F1MWL1	Cellular protein metabolic process	Actin cytoskeleton	Hydrolase activity	0.000	1.545
57	Uncharacterized protein	E1B7E1	Regulation of developmental process	Intracellular organelle	Protein binding	0.027	1.546
58	Band 4.1-like protein 5	Q58CU2		Cytoplasm; membrane	Protein binding	0.011	1.555
59	Myosin-1	Q9BE40	Metabolic process	Intracellular organelle	Binding	0.027	1.558
60	Dolichyl-diphosphooligosaccharide-protein glycosyltransferase subunit 1	A6QL95	Cellular biosynthetic process	Endoplasmic reticulum	Catalytic activity	0.027	1.560
61	DPYSL5 protein	A8E641	Developmental process	Cytoplasm	Hydrolase activity	0.000	1.560
62	MOB kinase activator 3A	Q58D63		Intracellular	cation binding	0.001	1.561
63	Store-operated calcium entry-associated regulatory factor	Q08E24	Regulation of transport	Intracellular organelle	–	0.041	1.565
64	Phosphoribosyl pyrophosphate synthase-associated protein 1	Q08DW2	Compound metabolic process	–	Transferase activity	0.005	1.578
65	Uncharacterized protein	F1MHH5	Cell–cell adhesion	Membrance	Signaling receptor activity	0.038	1.580
66	Nuclear speckle-splicing regulatory protein 1	F1MMV5	Metabolic process	Nucleus	Binding	0.002	1.583
67	SF3B2 protein	A4FV01	–	Intracellular	Nucleic acid binding	0.027	1.585
68	Guanine nucleotide-binding protein, beta-1 subunit	A7E3V7	Cellular response to organic substance	Membrane part	Hydrolase activity	0.004	1.604
69	Transcription factor MafF	A7YY73	Organ development	Intracellula	Nucleic acid binding	0.001	1.610
70	Sorting and assembly machinery component 50 homolog	G3MZZ3	Protein complex biogenesis	Integral component of membrane	–	0.001	1.611
71	Low-density lipoprotein receptor	P01131	Lipid metabolic process	Plasma membrane raft	–	0.003	1.612
72	MKK3 protein	A4IFH7	Inflammatory response	Intracellular	Protein kinase activity	0.002	1.619
73	Fibroblast growth factor	A6QPP3	Single-organism developmental process	Plasma membrane	Kinase regulator activity	0.029	1.619
74	Uncharacterized protein	E1BIU0	Cellular protein localization	Membrane	Binding	0.000	1.630
75	Uncharacterized protein (Fragment)	E1BJL9	Regulation of immune system process	Membrane	Cytokine receptor binding	0.025	1.634
76	AP-2 complex subunit beta	P63009	Intracellular protein transport	Membrane	Transporter activity	0.035	1.639
77	Uncharacterized protein	F1MPF7	Developmental process	Extracellular organelle	Binding	0.001	1.653
78	Scavenger receptor cysteine-rich type 1 protein M130	P85521	Inflammatory response	Membrane	Receptor activity	0.000	1.658
79	ALG2 protein	A4FUG6	Protein metabolic process	Cytoplasmic part	Protein binding	0.000	1.660
80	Collagen alpha-2(XI) chain	F1MRP6		Extracellular region	Ion binding	0.001	1.668
81	Uncharacterized protein	E1BMF2	Protein metabolic process	Extracellular region	Peptidase activity	0.002	1.677
82	Uncharacterized protein	F6R9F1	Cellular response to stimulus	Intracellular	Binding	0.000	1.679
83	Radial spoke head protein 3 homolog	A8E4N3	–	–	–	0.031	1.680
84	Cysteine and glycine-rich protein 2	Q32LE9	Developmental process	Intracellular	Ion binding	0.005	1.683
85	Arf-GAP domain and FG repeat-containing protein 1	Q2TA45	Developmental process	Membrane-bounded vesicle	Enzyme regulator activity	0.035	1.686
86	TACC3 protein	A6QL93	Response to stimulus	Intracellular organelle	Binding	0.000	1.687
87	Myristoylated alanine-rich C-kinase substrate	P12624		Cytoplasm	Protein binding	0.006	1.697
88	ER lumen protein-retaining receptor 1	P33946	Regulation of transport	Cytoplasmic part	Peptide binding	0.005	1.705
89	Kelch-like protein 9	F1MXI0	Metabolic process	Intracellular part	Transferase activity	0.033	1.713
90	Wiskott-Aldrich syndrome protein family member 2	A2VDK6	Cell migration	Extracellular region part	Protein binding	0.006	1.717
91	Cytosolic carboxypeptidase 3	G3N121	Protein metabolic process	Cytoplasm	Binding	0.034	1.717
92	Uncharacterized protein	F1MQ43	Regulation of metabolic process	Membrane	Enzyme binding	0.000	1.737
93	Collagen alpha-1(IV) chain	Q7SIB2	Single-organism process	Extracellular region|	Protein binding	0.002	1.763
94	Uncharacterized protein	E1BG99	Regulation of metabolic process	–	–	0.000	1.852
95	Uncharacterized protein	E1B9F3	Cellular developmental process	Cytoplasm	Protein binding	0.000	1.863
96	Uncharacterized protein	E1B7H4	Response to stress	Cytoplasm	Protein kinase activity	0.002	1.885
97	Protein phosphatase inhibitor 2	F1MTZ0	Regulation of signal transduction		Enzyme regulator activity	0.000	1.891
98	Fibronectin type 3 and ankyrin repeat domains protein 1	F1MCR5	–	Intracellular	–	0.000	1.901
99	Calcium/calmodulin-dependent protein kinase I	Q08DQ1	Biosynthetic process	Intracellular	Nucleoside binding	0.001	1.927
100	Nucleoredoxin	A6QLU8	Regulation of protein metabolic process	Cytoplasm	Antioxidant activity	0.004	1.949
101	Uncharacterized protein	F1MX40	Metabolic process	Nucleoside binding	Nucleotide binding	0.000	1.959
102	CA(2+)-dependent carbohydrate-binding protein	Q9TRL9	Regulation of developmental process	Nucleus	Binding	0.004	1.962
103	Uncharacterized protein	F1MJZ0	Regulation of system process	Membrane	Signaling receptor activity	0.001	1.970
104	PDZ and LIM domain protein 2	Q3T0C8		Cell junction	Cation binding	0.002	1.979
105	Uncharacterized protein	E1B7M1	Cellular developmental process	–	Exchange factor activity	0.022	1.998
106	Uncharacterized protein	F1MQI1	Cellular protein localization	Intracellular organelle	Receptor binding	0.021	2.085
107	Uncharacterized protein	F1MWF0	Regulation of protein metabolic process	Cytoplasmic vesicle	phospholipid binding	0.007	2.243
108	NCK adaptor protein 1	Q1LZB2	Regulation of signaling	Cell–cell junction	Protein kinase inhibitor activity	0.001	2.252
109	Sjoegren syndrome nuclear autoantigen 1 homolog	G3MWY9	Regulation of cellular process	Cytoplasm	Protein binding	0.000	2.254
110	Uncharacterized protein	E1BGM1	–	–	Ion binding	0.001	2.294
111	Pescadillo homolog	E1BM72	Primary metabolic process	Nucleus; membrane	Nucleic acid binding	0.028	2.434
112	Transcription elongation factor SPT5	A7YW40	Metabolic process	Nucleus; intracellular	Binding	0.000	2.604
113	Uncharacterized protein	F1MHA1	Biological regulation	Intracellular part	Transferase activity	0.000	3.021
114	Uncharacterized protein	F1MSV7	Regulation of secretion	Intracellular	Metal ion binding	0.008	3.075
115	Vesicle-associated membrane protein 3	G3X752	Regulation of secretion	cytoplasm	Binding	0.000	3.850
116	Uncharacterized protein	F1MJN7	–	–	Binding	0.0000	

*V/C* virus/control

### GO annotations of the DEPs

**Fig. 3:**
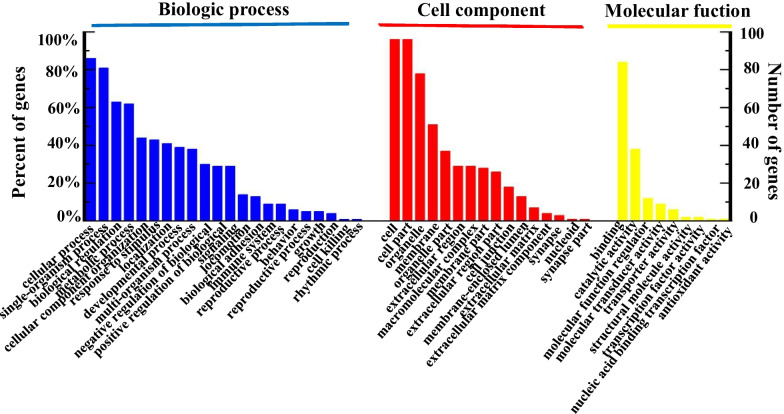
Gene ontology (biological process) analysis of the DEPs in BPIV3 groups vs. the control groups. The longitudinal coordinate-axis indicates the number of proteins for each GO annotation, the horizontal axis represents the GO annotations. The blue, biological processes categories of DEPs; red, categories of cell components; yellow, categories of the molecular functions

GO annotations for DEPs. The proteins were annotated into three major categories: biological process (BP), cellular component (CC), and molecular function (MF) (Fig. 3). The GO enrichment analysis in the biological process showed that the DEPs were significantly enriched in five processes, including single organism process, response to a stimulus, metabolic process, cell process, and biological regulation. The proteins involved in the biological regulation process were found most, followed by those involved in the stimulation response process. In this study, the proteins in the stimulation response process mainly included tyrosine phosphatase, signal transduction protein 1, Rab5 GDP/GTP conversion factor 1, interleukin-13 (IL-13), mitogen-activated protein kinase 7 (MAPK7), FOX transcription inhibitory factor 3 (Foxp3), calcium phosphate, protein tyrosine phosphatase protein receptor, MAP3K10, human telomerase reverse transcriptase, and SSNA1. IL-13 is the most important inflammatory factor that causes airway inflammation. It plays a key role in the occurrence of chronic airway inflammatory disease, which induces high secretion of mucus. Foxp3 is a member of the Fox transcription factor family that plays an important role in maintaining the immune function of the body [21]. The DEPs in BPIV3-infected MDBK cells may cause the initial cellular stress response. The precise role of these DEPs in the BPIV3 infection process need to be further investigated.

### Kyoto encyclopedia of genes and genomes (KEGG) pathway analysis of the DEPs

The KEGG pathway database is a collection map based on the molecular interaction pathways and cellular response networks. The DEPs were identified and mapped to six KEGG pathways, including metabolism, cellular processes, organismal systems, environmental information process, genetic information process, and disease pathways. The organismal systems and disease pathways were enrichment pathways, represented by 37 and 43 pathway groups, respectively.

**Fig. 4:**
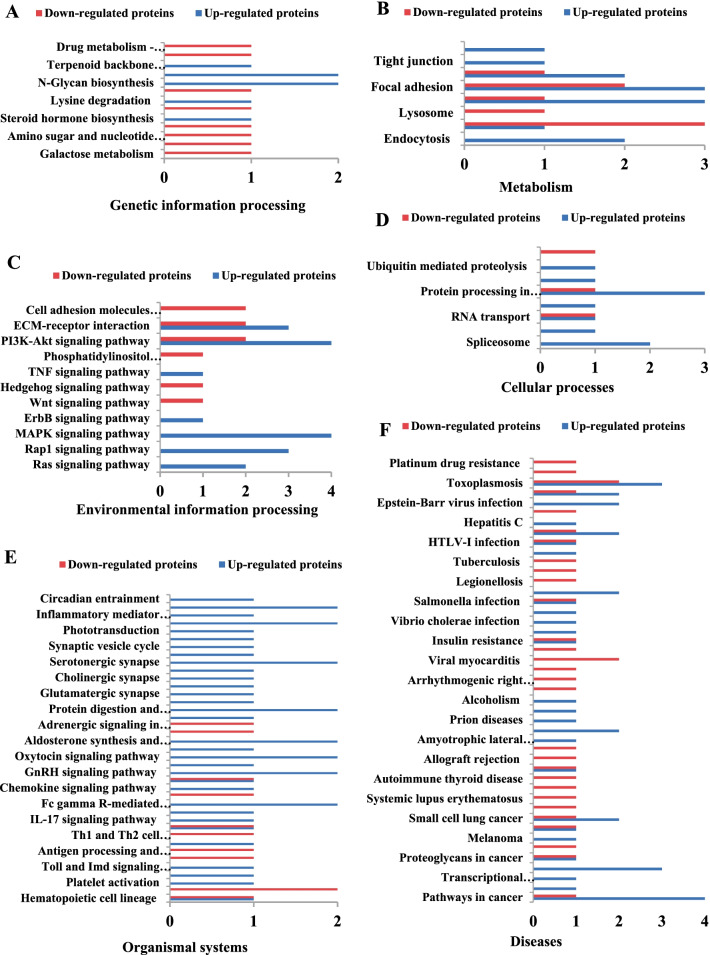
Analysis of the KEGG pathway of the differentially expressed proteins. **A** genetic information processing **B** Metabolism; **C** environmental information processing; **D** cellular processes; **E** organismal systems; **F** diseases

In the metabolic pathways, the DEPs participated in 13 pathways related to the metabolism of glucose, lipid, amino acid, and nucleotides (Fig. 4A). These pathways affect the metabolism of three major nutrients in cells. The cellular processes involved 10 pathways (Fig. 4B), including the Focal adhesion pathway and the Phagosome pathway, both of which were involved in the viral infection process. The integrin protein was the key protein in these two pathways. The lysosome pathway, phagosome pathway, and autophagy pathway were all involved in the autophagy process of virus infection. The environmental information involved 11 pathways, mainly focusing on the pathways of viral infection and the interaction of signal molecules (Fig. 4C). Among them, pI3K-Akt signaling pathway, MAPK signaling pathway, Ras signaling pathway and TNF signaling pathway have been proved to be related to virus infection. The annotated proteins in the category of genetic information processing played a role in the synthesis, transport, proteolysis, and spliceosome of cells (Fig. 4D). The annotated proteins in the organismal systems category were related to antigen processing and presentation, NOD-like receptor signaling, Toll-like receptor signaling, complement and coagulation cascades, and Th1 and Th2 cell differentiation pathway groups. These pathways were correlated with the immune response of the host to virus infection (Fig. 4E). The DEPs annotated in the disease category are shown in Fig. 4F. There are ten pathways clustering in infectious diseases, five of which are associated with viral infections.

According to the profiling of DEPs, a relatively large number of proteins were matched with the MAPK signaling pathway, including FGF13, ERK5, and MKK3. The KEGG pathway analysis revealed that MKK3 was involved in 14 pathways, indicating that MKK3 was a key regulatory protein during BPIV3 infection to MDBK cells (Table. 2).

### Validation of the selected proteins by real-time quantitative PCR (qRT-PCR)

**Fig. 5:**
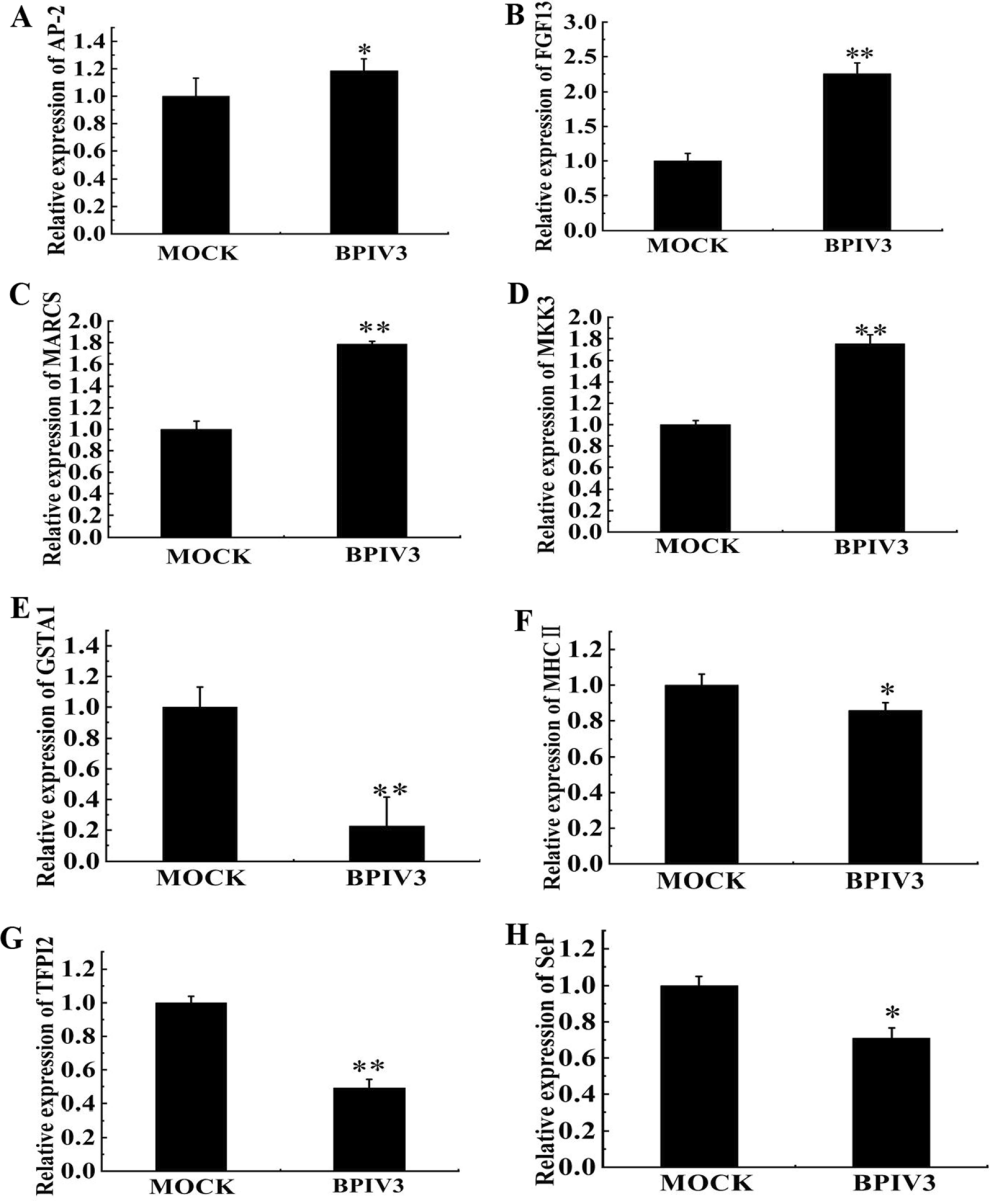
Real-time RT-PCR analysis of the DEPs in BPIV3-infected cells and controls. MDBK cells were infected with BPIV3 at MOI = 1 or mock-infected. The cells were collected at 24 hpi for real-time RT-PCR to analyze the relative expression of 8 differential expression genes. **A** AP-2; **B** FGF13; **C** MARCS; **D** MKK3; **E** GSTA1; **F** MHCII; **G** TFPI2; **H** SepP

To verify the DEPs identified by iTRAQ, the transcriptional levels of eight proteins were measured by qRT-PCR. In this study, the eight proteins were randomly selected for qRT-PCR. The four of them upregulated proteins included AP-2 complex subunit beta protein (AP-2), FGF13, myristoylated alanine-rich C-kinase substrate (MARCS), and MKK3 proteins. The other four downregulated proteins included MHC class II (MHCII), glutathione S-transferase (GSTA1), selenium protein P (SepP), and tissue factor pathway inhibitor (TFPI). As shown in Fig. 5, the expression levels of these genes were consistent with the iTRAQ results. The results of qRT-PCR further verified the reliability of the iTRAQ experiment.

### The effect of the p38 MAPK pathway on BPIV3 replication

#### BPIV3 infection activating the p38 MAPK pathway

The MAPK pathway plays various roles in intracellular signaling network. MKK3 and MKK6 are recognized as upstream kinases of p38. The results of proteomics analysis showed that the MKK3 level was significantly upregulated after BPIV3 infection (Table. 2). Virus infection is considered as an extracellular stimulant that can activate p38 MAPK pathway [22, 23]. It should be investigated whether BPIV3 infection activated the p38 MAPK pathway after MKK3 activation.

**Fig. 6:**
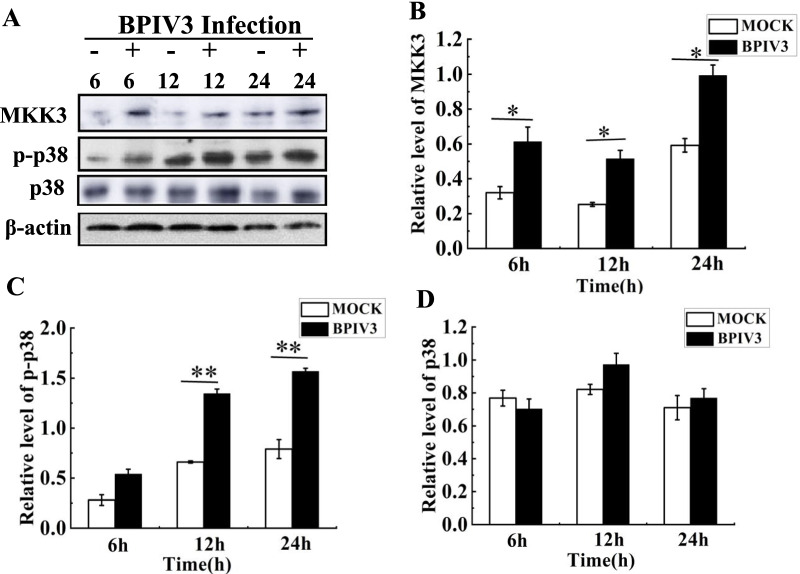
The p38 MAPK pathway was activated by BPIV3 infection. The MDBK cells were collected in mock-infected group or BPIV3-infected group (MOI = 1) from 6 to 24 h. MKK3, p38 phosphorylation and total amount of p38 were analyzed in whole-cell lysates by Western blot. The primary antibodies were the specific anti-phospho-p38 antibodies (mouse, 9216, CST, USA), anti-p38 antibodies (rabbit, 41666, CST, USA) and anti-MKK3 antibodies (rabbit, 5674, CST, USA), the second antibodies were goat anti-mouse and goat anti-rabbit IgG. β-actin probed with specific monoclonal antibody was served as internal control. Densitometry scans were conducted by ImageJ software (NIH, USA). Densitometry of the phospho-p38 band was normalized to p38, which was presented as fold change ± SEM compared with the mock-infected control defined as 1. These data were from three independent experiments. Significant differences compared with mock-infected control are denoted by *(P < 0.05), ** (P < 0.01). The Same densitometry analysis and statistical analysis were performed in the following experiments. **A** The protein expression in p38 MAPK pathway by Western blot; **B** Expression of MKK3; **C** Expression of p-p38; **D** Expression of p38

The expression of MKK3, p38, and phospho-p38 in BPIV3-infected cells was detected by western blotting assay. Cell samples were collected at 6, 12, and 24 h post BPIV3 infection. Compared to the mock group, the MKK3 expression levels were increased at different infection time points in the infected group. No change was observed in the p38 protein expression level, while the phospho-p38 expression level was significantly higher in the infected group than in the mock group at 12 h and 24 h after BPIV3 infection (Fig. 6). Thus, BPIV3 infection induced MKK3 activation and p38 phosphorylation. The MKK3 expression level was consistent with previous proteomics results, which further verified the reliability of proteomics analysis.

### The effect of inhibiting p38 MAPK activation on BPIV3 replication

To investigate whether the activation of the p38 MAPK pathway promotes BPIV3 proliferation, the cells were treated with SB202190, an inhibitor of the p38 MAPK pathway, at 1 h before infection. The MDBK cells were treated with SB202190 at concentrations of 1.25, 5, and 10 μM. Cell samples were collected at 24 h after infection (MOI = 1).

**Fig. 7:**
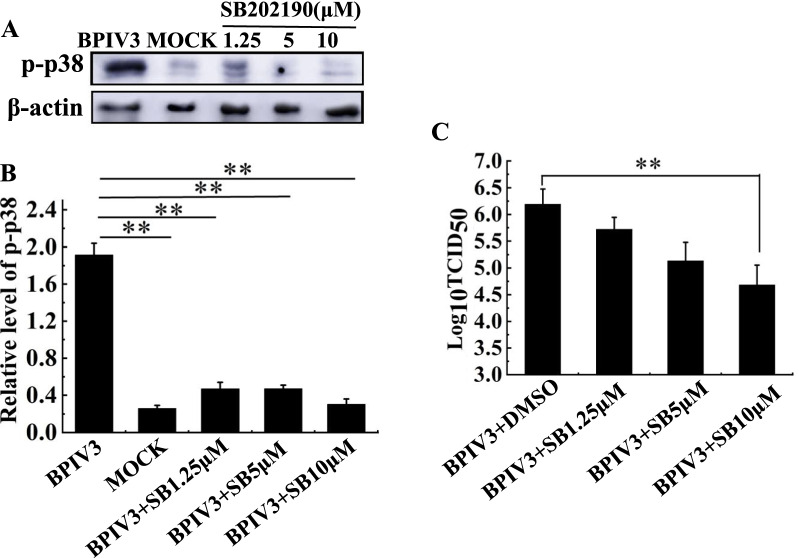
Inhibition of activation of the p38 pathway inhibits BPIV3 replication. The MDBK cells were treated with SB202190 at 1.25, 5, and 10 μM concentrations. After 1 h, BPIV3-infected cells were inoculated with MOI = 1. The cell samples were collected at 24 h after infection, and the following tests were performed. (A and B) SB202190 impact on p38MAPK phosphorylation. Cell samples were collected 24 h after infection, lysed with cell lysate, and the expression of phospho-p38 and β-actin in the samples was detected by Western-blot; (C)SB202190 impact on Bpiv3 TCID_50_. The cell supernatant was collected 24 h after infection, and the titer of the virus was detected by TCID_50_ assay. ** (P < 0.01)

The results are shown in Fig. 7. The BPIV3 infection induced the phosphorylation of p38. After treatment with the inhibitor SB202190, the expression level of p38 was significantly decreased in a dose-dependent manner, indicating that the phosphorylation of p38 was inhibited by SB202190 (Fig. 7A and B). The BPIV3 virus titer decreased by 1.8 logTCID_50_/mL after treatment with 10 μM SB202190, indicating that the p38 MAPK pathway participates in the replication of BPIV3 (Fig. 7C). The results showed that SB202190 could inhibit the proliferation of BPIV3. Thus, BPIV3 activated the p38 MAPK signaling pathway that is involved in its replication.

## Discussion

iTRAQ LC-MS/MS is a powerful analytical tool for quantitative proteomics analysis that has been widely used in many studies [24–27]. Gray et.al used 2D gel electrophoresis proteomic to investigate in vitro cellular responses during BPIV3 infection [28]. In the present study, we first applied the iTRAQ LC-MS/MS approach to determine the profiles of DEPs in MDBK cells infected with BPIV3 at 24hpi. A total of 116 DEPs were identified at 24 h after infection. On the basis of GO analysis, the DEPs were classified into 19, 11, and 9 categories for biological processes, cellular components, and molecular functions, respectively (Fig. 3). The pathway analysis identified the pathways based on the number of DEPs (Fig. 4). These data could provide a basis for understanding the pathogenetic mechanisms of BPIV3 infection.

The results showed that the PI3K-Akt signaling pathway and the MAPK signaling pathway play important roles in the progression of BPIV3 infection. According to the profiles of DEPs in these two signaling pathways, only ITGB3 was downregulated, while the remaining proteins were upregulated. Interestingly enough, the number of matched proteins in the MAPK signaling pathway was relatively large, including FGF13, ERK5, and MKK3. The KEGG pathway analysis further indicated that MKK3 was involved in 14 pathways, which suggested that MKK3 is a key regulatory protein during BPIV3 infection. Previous studies have shown that the MAPK signaling pathway is a target of respiratory viruses, which regulates various stages of the infection process [29, 30].

The MAPK cascade plays various roles in intracellular signaling network pathways. MKK3 and MKK6 are recognized as upstream kinases of p38 that can directly phosphorylate tyrosine and serine/threonine residues to activate p38 [31]. Viral infection is thought to be an extracellular stimulant that activates this pathway. Immunohistochemical detection showed that the phosphorylation level of p-ERk1/p-p38 in the lungs of sheep infected with infectious salmon anemia virus (ISAV) was significantly increased compared to that in healthy sheep [22]. In our proteomics study, the MKK3 level was significantly upregulated at 24 h after BPIV3 infection compared to that in the control group. Therefore, we detected the protein expression level in the p38 MAPK pathway after BPIV3 infection.

First, we investigated whether BPIV3 infection activates the p38 MAPK pathway. The results showed that BPIV3 induced the phosphorylation of p38 after infection. Compared to the control group, the phosphorylated p38 expression was significantly increased after 6 h of BPIV3 infection, demonstrating that BPIV3 could induce the activation of the p38 MAPK pathway in the early stage of infection.

Multiple extracellular stresses activate the MKK3-p38 MAPK cascade, including specific antigens, proinflammatory cytokines, ultraviolet light, heat shock, and other stress responses [32]. In accordance with the results of the mechanism of Coxsackie virus activation of p38 MAPK, MKK3-p38 MAPK was temporarily activated in the early stage of infection [33]. The same results were found in our study, MKK3-p38 MAPK was activated at 6 h post BPIV3 infection. As the BPIV3 infection was gradually prolonged, the phosphorylation of p38 MAPK was more significantly increased at 24 h after infection. In the late stage of infection, p38 was still continuously activated, which was speculated to be due to the release of proinflammatory cytokines induced by BPIV3 infection. These released proinflammatory cytokines bound to the receptor further enhanced the activation in the p38 MAPK pathway [34, 35].

Many studies have shown that p38 is required for the replication of viruses. The activation of the MAPK pathways by viruses such as stimulates the JNK and p38 MAPK pathways to promote the release of virions [32]. In porcine reproductive and respiratory syndrome virus infection, the virus replication was inhibited after inhibition of the JNK and p38 pathways [36]. The same results were noted in PEDV infection [37]. To detect the role of the p38 MAPK pathway in BPIV3 replication, virus titer was analyzed. We found the inhibitor SB202190 significantly inhibited BPIV3 replication in a dose-dependent manner. It was also found that p38 expression was inhibited after treatment with SB202190. Compared with the untreated group, the virus titer was significantly decreased in the inhibitor treatment cells. These results revealed that the activation of the p38 MAPK pathway facilitated replication of BPIV3.

## Conclusion

In this study, DEPs in BPIV3-infected MDBK cells were identified and quantitatively analyzed by iTRAQ and LC-MS-based proteomics analysis. Most of the DEPs were proteins related to inflammatory response, immune response, and lipid metabolism. Although many significantly up- or downregulated proteins and pathways are closely related to the symptoms or pathological responses to BPIV3 infection, further functional investigations are required to understand the pathogenic mechanisms and molecular responses of host cells to BPIV3 infection.

The results of the present study indicated that BPIV3 infection activates the p38 MAPK pathway, which is essential for its replication. Proteomics and western blot analyses showed that BPIV3 infection activated the p38 MAPK signaling pathway. Our future research will focus on which step of virus replication is affected by p38 activation.

## Data Availability

Not applicable.

## Abbreviations

BPIV3: Bovine parainfluenza virus type 3
BRDC: Bovine respiratory disease complex
iTRAQ: Isobaric tags for relative and absolute quantitation analysis
DEPs: Differentially expressed proteins
TGEV: Transmissible gastroenteritis virus
PEDV: Porcine epidemic diarrhea virus
BRSV: Bovine respiratory syncytial virus
DMEM: Dulbecco’s modified Eagle’s medium
FBS: Fetal bovine serum
CPE: Cytopathic effect
GO: Gene ontology
IFA: Indirect immuno- fluorescent assay
MOI: Multiplicity of infection
IL-13: Interleukin-13
MAPK7: Mitogen-activated protein kinase 7
Foxp3: FOX transcription inhibitory factor 3
MARCS: Myristoylated alanine-rich C-kinase substrate
MHCII: MHC class II
GSTA1: Glutathione S-transferase
SepP: Selenium protein P
TFPI: Tissue factor pathway inhibitor
